# What the radiologist should know about the role of interventional radiology in urology

**DOI:** 10.1590/0100-3984.2018.0035

**Published:** 2019

**Authors:** Tiago Kojun Tibana, Vinícius Adami Vayego Fornazari, Walberth Gutierrez Junior, Edson Marchiori, Denis Szejnfeld, Thiago Franchi Nunes

**Affiliations:** 1 Hospital Universitário Maria Aparecida Pedrossian da Universidade Federal de Mato Grosso do Sul (HUMAP-UFMS), Campo Grande, MS, Brazil.; 2 Escola Paulista de Medicina da Universidade Federal de São Paulo (EPM-Unifesp), São Paulo, SP, Brazil.; 3 Universidade Federal do Rio de Janeiro (UFRJ), Rio de Janeiro, RJ, Brazil.

**Keywords:** Radiology, interventional, Urology, Fluoroscopy, Tomography, X-ray computed, Ultrasonography, Magnetic resonance imaging, Radiologia intervencionista, Urologia, Fluoroscopia, Tomografia computadorizada, Ultrassonografia, Ressonância magnética

## Abstract

Interventional radiology has been constantly developing in terms of the techniques, materials, and methods of intervention. It interacts with all areas of medicine, always with the ultimate goal of ensuring the well-being of patients. Advances in imaging techniques, especially in the last two decades, have led to a paradigm shift in the field of urological imaging interventions. Many urologic diseases that were previously treated only surgically can now be effectively managed using minimally invasive image-guided techniques, often with shorter hospital stays and requiring only local anesthesia or conscious sedation.

## Abstract (pt)

A radiologia intervencionista vem se desenvolvendo constantemente por meio de técnicas, materiais e métodos de intervenção. Interage com todas as áreas da medicina, sempre visando, como objetivo final, o bem-estar dos pacientes. Os avanços das técnicas de imagem, especialmente nas últimas duas décadas, levaram a uma mudança de paradigma no campo das intervenções guiadas por imagens na urologia. Muitas doenças urológicas que eram tratadas somente cirurgicamente, podem agora ser manejadas efetivamente usando técnicas minimamente invasivas guiadas por imagem, muitas vezes com redução do tempo de internação e utilizando apenas anestesia local ou sedação consciente.

## INTRODUCTION

Image-guided interventions performed by interventional radiologists have changed the management of various abdominal conditions, urologic conditions in particular^(^^1^^-^^5^^)^. The number of potential applications of such interventions is growing because of their minimally invasive nature, the minimal morbidity associated with the procedures, and the fact that their use can shorten hospital stays. Some traditional diagnostic procedures have evolved to become state-of-the-art therapeutic techniques, which include a wide range of vascular and nonvascular applications. Interventional radiology techniques continue to play an important role in drainage procedures, urolithiasis management, dilatation of the renal pelvis, tumor ablation, and the treatment of renovascular diseases. Multiple imaging modalities are used for these purposes, primarily fluoroscopy, ultrasonography, computed tomography, magnetic resonance imaging, and digital subtraction angiography. It is important that a close multidisciplinary collaboration be maintained among urologists, nephrologists, and interventional radiologists^(^^6^^)^. This pictorial essay examines various aspects of interventional uroradiology.

## OBSTRUCTIVE UROPATHY

**Figure 1:**
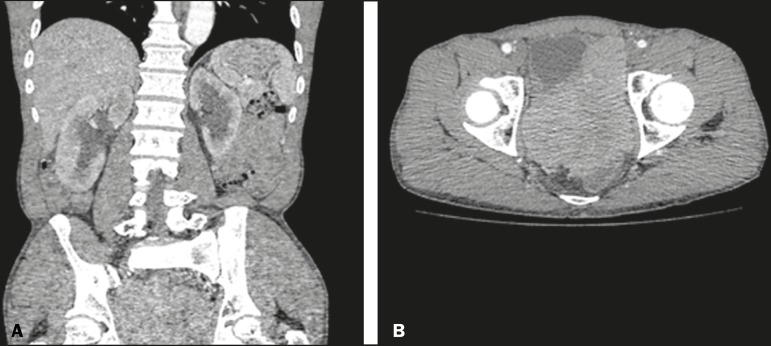
Contrast-enhanced coronal and axial computed tomography of the abdomen (**A** and **B**, respectively), showing marked bilateral hydronephrosis, caused by a mass (sarcoma) in the prostate gland, invading both ureteral orifices and making cystoscopic JJ stenting impossible.

**Figure 2:**
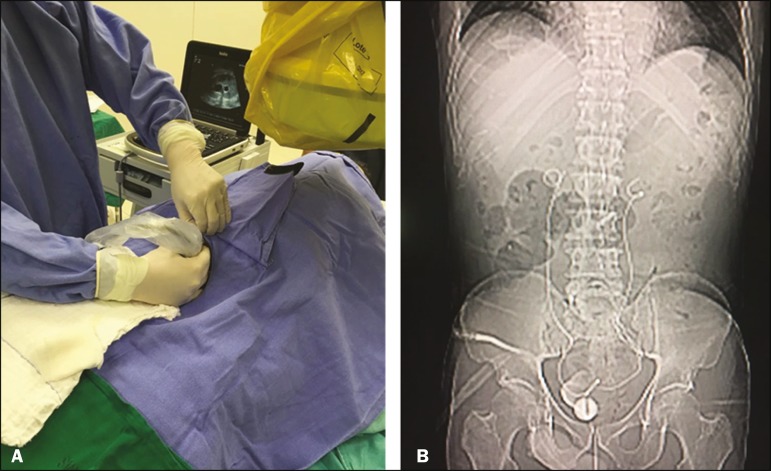
Planning of percutaneous JJ stenting with ultrasound of the urinary tract in the hemodynamics room (**A**). X-ray showing adequate positioning of both ends of the JJ stent after the procedure (**B**).

Interruption of the normal flow of urine is one of the most common causes of acute and chronic renal failure (Figure 1), requiring the use a procedure that has the objective of restoring physiological urine flow. Drainage of the urinary tract can be accomplished by a number of techniques and devices, including retrograde or anterograde percutaneous insertion of the double J catheter (JJ stenting), percutaneous nephrostomy, and, more recently, the use of ureteral prostheses. Percutaneous JJ stenting restores physiological urinary drainage without the need for an external catheter (Figure 2). Although it has a high success rate, the techniques involved have not been widely disseminated^(^^7^^)^.

One major advance was the development of metallic mesh stents. Such stents, including expandable balloons, as well as self-expanding, thermoplastic, and coated stents, have been used in the treatment of various conditions, such as benign and malignant ureteral stenoses^(^^8^^)^.

The advantages of interventional radiology procedures include a lower risk of complications and the fact that they can be performed under local anesthesia and sedation, minimizing the risks of adverse post-anesthesia events in comparison with general anesthesia, especially in critically ill patients.

## MACROSCOPIC HEMATURIA

Hematuria, which can be microscopic or macroscopic, is a common medical problem, identified on screening tests in up to 18% of asymptomatic individuals. Various pathological renal vascular processes have been associated with hematuria, including aneurysm or pseudoaneurysm of the renal artery, fibromuscular dysplasia, nutcracker syndrome, arteriovenous malformation, vasculitis, thrombosis of the renal artery or vein, and neoplasia^(^^9^^)^.

**Figure 3:**
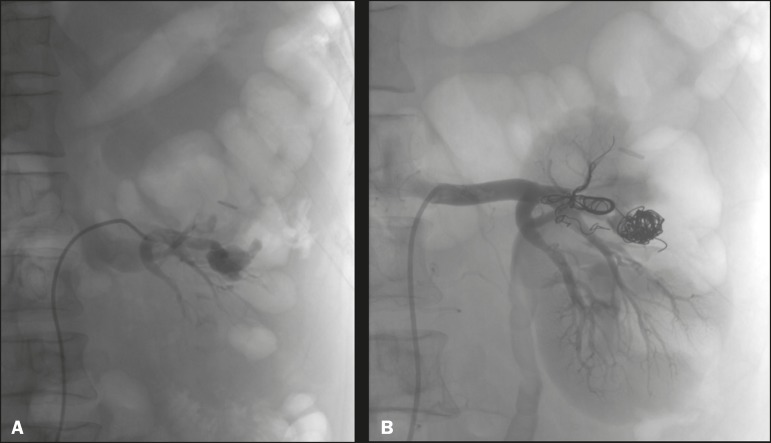
Renal angiography showing pseudoaneurysm after partial nephrectomy (**A**), evolving to macroscopic hematuria. Embolization with vascular coils (**B**).

**Figure 4:**
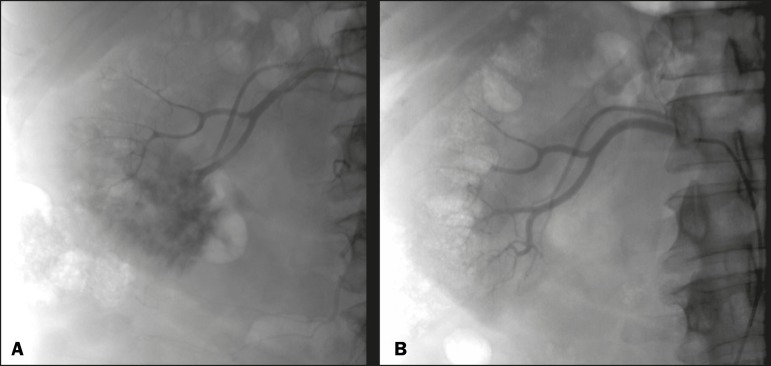
Patient with macroscopic hematuria who had been diagnosed with renal neoplasia that was refractory to treatment and were not candidates for surgery. Renal angiography showing a vascularized, poorly delimited tumor (**A**). The patient was treated with embolization. Follow-up renal angiography showing regression of the findings, indicating the success of the procedure (**B**).

**Figure 5:**
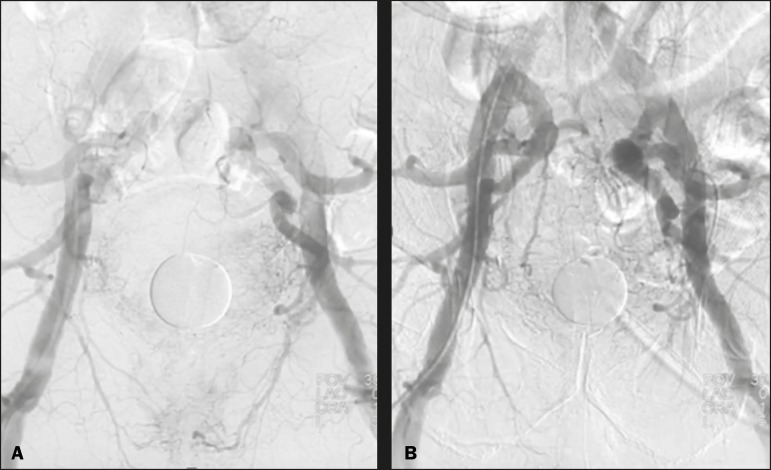
Pre- and post-treatment angiography with selective embolization (**A** and **B**, respectively), showing good results in patients with previously resected prostate cancer, presenting hematuria due to locoregional tumor recurrence.

Embolization is a minimally invasive procedure, in which the lumen of a vessel is occluded by embolization material, with reported success rates of up to 90%^(^^6^^)^. Indications include, but are not limited to, persistent hematuria resulting from a pseudoaneurysm (Figure 3), post-biopsy arteriovenous fistula, renal tumors (Figure 4), recurrence of prostate cancer (Figure 5), vascular malformations, and surgical or accidental trauma. The materials typically used for this purpose are collagen hemostatic sponges and vascular coils^(^^6^^)^.

**Figure 6:**
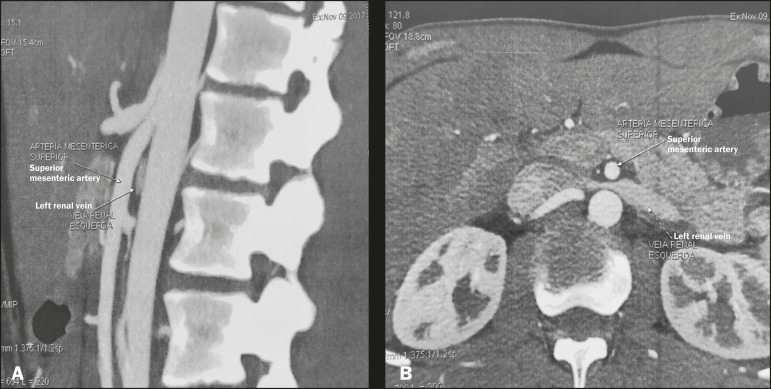
Sagittal and axial computed tomography angiography (**A** and **B**, respectively), showing compression of the left renal vein between the aorta and the superior mesenteric artery (nutcracker syndrome) in a patient with varicocele.

**Figure 7:**
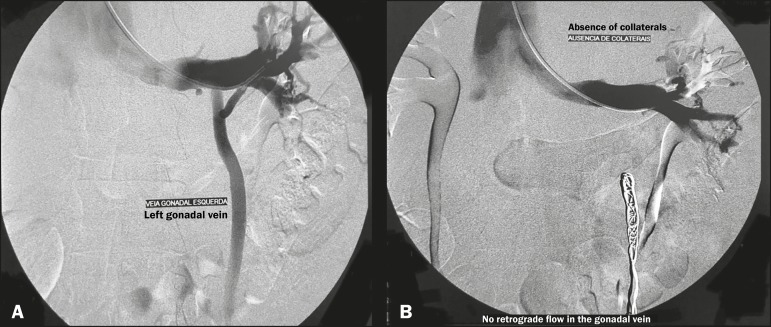
Angiography showing endovascular treatment with dense foam and fibered coils (**A**,**B**) in a patient with nutcracker syndrome.

Nutcracker syndrome is characterized by a set of signs and symptoms secondary to compression of the left renal vein (Figure 6). The compression point occurs most commonly between the superior mesenteric artery and the aorta. Until recently, the treatment was restricted to conventional open surgery, although it can now be performed with less invasive endovascular methods^(^^10^^)^, as depicted in Figure 7.

## RETROPERITONEAL HEMATOMA

Spontaneous ruptures of the renal parenchyma, resulting in retroperitoneal hematoma, are caused by tumors in 50-60% of cases. Angiomyolipoma and renal adenocarcinoma account for two thirds of all such tumors. In addition to the rich vascularization of these tumors, there are structural changes in the vessel walls that predispose them to rupture. Treatment approaches include superselective arterial embolization, tumor enucleation, and, in some cases, nephrectomy. Embolization should be considered the initial treatment of choice in symptomatic patients^(^^11^^)^.

## TREATMENT OF TUMORS

**Figure 8:**
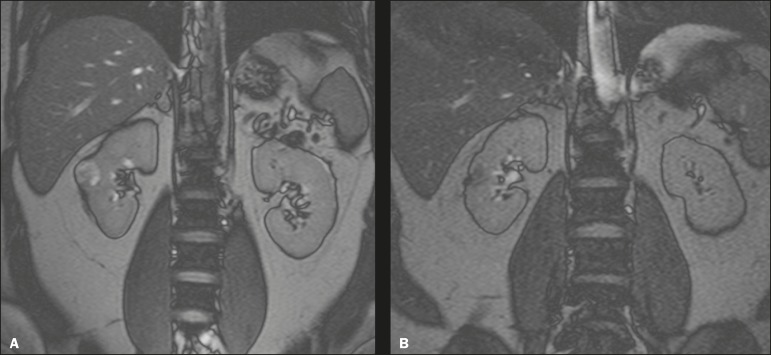
In-phase gradient-echo coronal magnetic resonance imaging of the abdomen, showing renal cell carcinoma in the right mid-kidney (**A**). Follow-up magnetic resonance imaging after percutaneous ablation, showing regression of the tumor (**B**).

**Figure 9:**
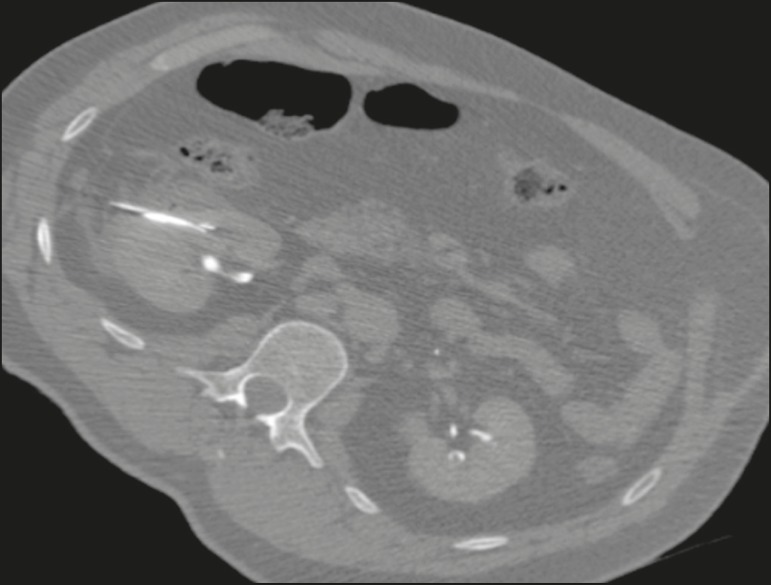
Axial computed tomography of the abdomen, showing percutaneous ablation of a renal cell carcinoma.

Among the many options for the treatment of benign and malignant tumors affecting multiple organs, image-guided percutaneous ablation is become increasingly more widely accepted^(^^4^^)^, especially in patients who are at high surgical risk (Figure 8). There are currently a number of thermal and non-thermal ablation modalities available^(^^9^^)^, including radiofrequency ablation, microwave ablation, cryoablation, high-intensity focused ultrasound, laser ablation, irreversible electroporation, chemical ablation (with ethanol or acetic acid), and brachytherapy (Figure 9).

In patients with recurrent prostate cancer, who typically present with hematuria, selective embolization is safe and should be considered the treatment of choice, because it usually precludes the need for emergency surgery in such critically ill patients^(^^12^^)^.

## TREATMENT OF SECONDARY HYPERTENSION

**Figure 10:**
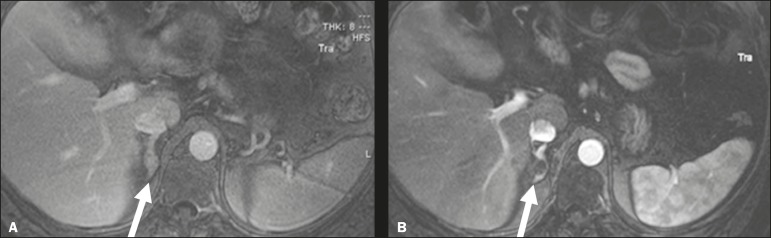
Primary hyperaldosteronism in a patient diagnosed with functional adrenal adenoma in a single adrenal gland (arrow), visualized on contrast-enhanced fat-saturated T1-weighted magnetic resonance imaging of the abdomen (**A**). Percutaneous ablation, performed as an alternative to laparoscopic treatment, provided improvement in the clinical and imaging parameters. Follow-up magnetic resonance imaging showing the success of the procedure (**B**).

The prevalence of secondary hypertension ranges from 3% to 5%. Among the various causes are renovascular disease and primary hyperaldosteronism resulting from a unilateral aldosterone-producing adenoma (Figure 10).

Renal artery stenosis is found in 2% of the general population and in 40% of the population at high risk for cardiovascular disease. For hemodynamically significant stenosis, characterized by a trans-stenotic pressure gradient ≥ 20 mmHg, the preferred treatment is recanalization, which involves balloon angioplasty, with or without stenting. The technical success of renal angioplasty depends on the nature of the stenosis and the underlying causal factor, ranging from 40% in stenosis caused by arteriosclerosis to 90% in that caused by fibromuscular dysplasia. The success rate for angioplasty is higher with stenting than without^(^^6^^)^.

**Figure 11:**
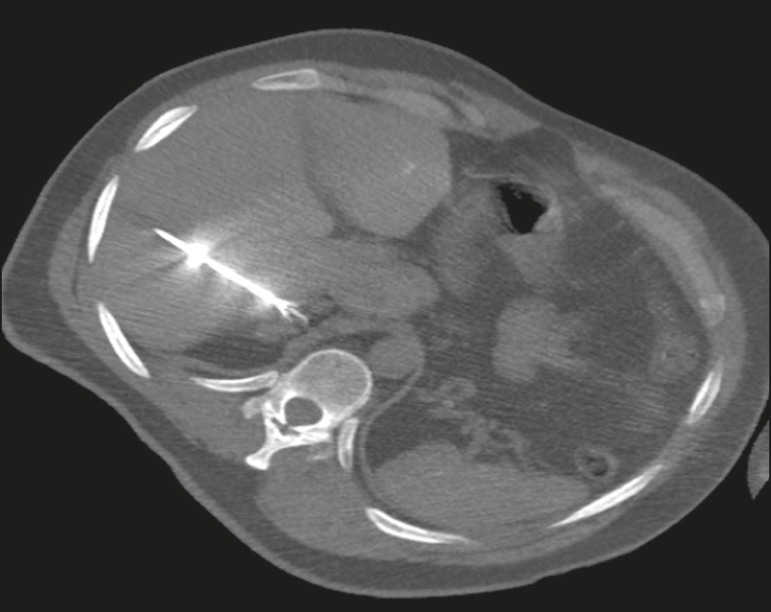
Axial computed tomography of the abdomen, showing percutaneous ablation of an adrenal adenoma.

The estimated prevalence of aldosterone-producing adenoma in hypertensive patients with incidentalomas is 1.6-5.0%. In young patients with hypertension, the clinical suspicion of aldosterone-producing adenoma is increased in the presence of refractory hypertension, hypokalemia, or a positive family history. Although the conventional treatment for functional adrenal adenomas is surgical resection, percutaneous ablation has been shown to be a good option for laparoscopic treatment^(^^13^^,^^14^^)^, as shown in Figure 11.

## VASCULAR EMBOLIZATION

In patients complaining of low back pain and infertility who present with varicocele, vascular embolization has been shown to be an excellent treatment modality. The approach is either jugular or femoral. After diagnostic angiography, the veins are embolized. The clinical results of technically successful percutaneous internal sperm embolization are similar to those of surgical treatment^(^^1^^)^.

Priapism can be the result of a variety of factors and is often treatable. During the initial evaluation, it is essential to differentiate the causes of high and low flow, given that the pathophysiology and treatment are different. Selective embolization of an arteriocavernosal fistula with absorbable or nonabsorbable materials is effective, producing better results, as well as having a lower rate of complications, than does surgical ligation^(^^15^^)^.

## CONCLUSION

Image-guided interventional radiology procedures are now part of contemporary urology practice. They are minimally invasive treatment strategies with low morbidity rates. The expectation is for even greater expansion, with new applications and imaging techniques. It is important that non-interventional radiologists and urologists become familiar with the variety of interventional radiology procedures that can be performed, so that an ever greater number of patients can benefit.
